# Pros and cons of surgical versus conservative management for head and neck paraganglioma: a real-world data analysis

**DOI:** 10.1007/s12020-025-04167-1

**Published:** 2025-01-26

**Authors:** L. Canu, L. Zanatta, C. Sparano, R. Santoro, G. Mannelli, S. Zamengo, B. Jance, F. Amore, T. Ercolino, M. Mannelli, M. Maggi, E. Rapizzi

**Affiliations:** 1 Department of Experimental and Clinical Biomedical Sciences “Mario Serio”, University of Florence, Florence, Italy; 2 Endocrinology Unit, Careggi University Hospital, Florence, Italy; 3 Centro di Ricerca e Innovazione sulle Patologie Surrenaliche, AOU Careggi, Florence, Italy; 4 ENS@T Center of Excellence, Florence, Italy; 5 Department of Experimental and Clinical Medicine, University of Florence, Florence, Italy; 6 Dept. of Experimental and Clinical Medicine, University of Florence, Florence, Italy

**Keywords:** Head and neck paragangliomas, Surgery, Radiotherapy, Wait and see, Otolaryngologist visit

## Abstract

**Purpose:**

To compare functional deficits associated to surgery with those caused by the growth of the head and neck paragangliomas (HNPGLs).

**Methods:**

72 patients with HNPGLs were included. Patients were divided in group A (49 patients undergoing surgery) and group B (23 patients following a wait and see approach). Each patient was subjected to clinical evaluation and genetic testing. The presence of functional neurological deficits in speech or swallowing and quality of life were assessed via a dedicated otolaryngologist visit, three posted questionnaires (VHI, DHI, and MDADI), and the European Organization for Research and Treatment of Cancer Quality of Life Questionnaire-H&N35.

**Results:**

Thirty-four patients from group A (69.4%) and 18 from group B (78.2%) agreed to fill out the posted questionnaires. Eighteen patients from group A (36.7%) and 10 from group B (43.5%) underwent a dedicated otolaryngologist visit. A significant difference between group A and B was observed in the VHI (p = 0.001) and DHI scoring (p = 0.020), and at the otolaryngologist visit (mild neurological disabilities, p = 0.007). Patients with familial forms presented multiple HNPGLs (p = 0.011), multiple secreting lesions (p = 0.010) and underwent surgery more times for HNPGLs (p = 0.009) and for both HNPGLs and sympathetic PGLs (p = 0.015). ROC curve analysis suggests that surgery in carotid body tumors >34 mm may be more frequently associated with nerve injury.

**Conclusion:**

The management of HNPGL patients remains challenging for clinicians. This preliminary study seems to suggest that surgery still represents the first choice for patients with small lesions. An accurate clinical evaluation is mandatory to avoid non-resolving surgery and possible neurovascular long-term complications.

## Introduction

Paragangliomas (PPGLs) are rare, highly vascularized tumors. Head and neck paragangliomas (HNPGLs) arise from the parasympathetic ganglia of the head and neck and are usually non-functioning [1], at variance with thorax-abdomen sympathetic PGLs that release catecholamines [2, 3]. HNPGLs more frequently localize in the carotid body, followed by the jugular bulb, tympanic plexus, vagal nerve, and larynx [4].

Usually, HNPGLs are non-aggressive tumors with a metastatic risk ranging from 2 to 19% depending on the localization [4]. There are no validated biochemical or molecular parameters to predict malignancy, defined as the presence of metastasis in tissues devoid of chromaffin cells [5]. HNPGLs may occur sporadically or be associated with germline variants in one of the many susceptibility genes.

The majority of familial HNPGLs (50–70%) [6] are associated with *SDHD* and *SDHB* germline variants, followed by *SDHC*, *VHL*, *SDHAF2*, *SDHA*, *TMEM127*, *MAX*, *RET*, and *NF1* [4, 7]. *SDHB-*associated lesions are at highest risk for metastases [8, 9]. Clinically, carotid body and vagal PGLs are painless, slowly growing neck masses along the sternocleidomastoid muscle, at the upper part of the vascular-nervous bundle of the neck, eventually causing tonsillar bulging. With their growth, they can also involve inferior cranial nerves, causing speech and swallowing disabilities in 10% of patients. Differently, tympanic PGLs are usually symptomatic for pulsatile tinnitus or hearing loss. Their growth may lead to other cranial nerves dysfunction or skull-base bone destruction. Laryngeal PGLs can cause shortness of breath, hoarseness, and stridor [1–4, 10].

Carotid body tumors have been classified by Shamblin according to the grade of involvement of the carotid vessels [11] (Table 1). Later on, this classification was modified by Luna-Ortiz to predict post operative morbidity [12].

**Table 1:** Shamblin’s classification of carotid body tumors

Type I	Small, localized tumor that is not adherent to the carotid vessels. May cause splaying of the carotid bifurcation.
Type II	Bigger tumors (>4 cm) adherent to or partially surrounding the carotid vessels, close to the nervous structures.
Type III	Bigger tumors, intimately surrounding or encasing the carotid vessels. They may reach the cranial base.

Jugulo-tympanic PGLs have been classified by Fisch to describe the best surgical approach based on their dimension and involvement of temporal bone [13].

Historically, surgery with or without preoperative embolization [1, 14] was the main treatment of HNPGLs, and it was the first choice in the case of *SDHB* HNPGLs, due to the high metastatic risk [2]. Albeit successful in 90–97% of cases and associated with a very low mortality rate (0–2.7%), surgery is often responsible for important side effects, including hemorrhagic, cerebrovascular, and neurological complications, such as blood pressure instability or stroke, and post-operative cranial nerve palsy, finally leading to deglutitiondysfunctions, speech problems, and hearing loss. Post-operative complications can be alleviated thanks to rehabilitation, but they impact quality of life and have social costs [10, 15].

Radiotherapy can be considered a primary treatment or adjuvant therapy after subtotal resection. The radiotherapeutic options are conventional fractionated external-beam radiation therapy (EBRT) or stereotactic radiosurgery (SRS). These techniques demonstrated local tumor control in 88–100% of cases, with rare evidence of toxicity [10].

A conservative “wait and see” approach has been proposed by many authors such a strategy could prevent treatment-associated complications in many patients, considering surgical intervention only in cases of tumor growth or new cranial nerve deficit [16, 17].

This study aims to compare any functional deficits associated with surgery with those associated with the growth of the tumor, by comparing the rate of adverse events affecting those undergoing surgery for HNPGLs to those undergoing a watchful waiting approach.

## Materials and methods

### Study design

This is a retrospective cross-sectional study.

### Patients

We enrolled 72 outpatients affected by HNPGLs between January 2000 and May 2022. All participants gave written consent for the enrolment (ENS@T protocol 59/11, version 1.3). All of them were visited and evaluated in the Endocrinology Unit of Azienda Ospedaliero Universitaria (AOU) Careggi.

The inclusion criteria were the occurrence of one or more HNPGLs and consent to being interviewed. The exclusion criteria were pre-existing neurological disorders or lesions, end of the post-surgery follow-up in patients surgically treated, and head and neck surgery due to reasons other than HNPGL.

The diagnosis of HNPGL was made by imaging (highly vascular mass in the head-neck region on CT or MRI and positive Octreoscan or Gallium PET) and/or confirmed by histology for surgically removed lesions.

We collected all the relevant clinical information, including gender, age at diagnosis, number, dimensions, localization and grading of the HNPGL), genetic variants, kind of detection (incidentally detected, familial screening), associated symptoms at diagnosis, other non-surgical treatment, associated secreting PGLs, and new HNPGL after surgical treatment.

### Genetic analysis

A genetic test was offered to all patients to find any germline variants in DNA extracted from peripheral blood leucocytes. In patients enrolled before 2017, mutations of *SDHB, SDHC, SDHD, VHL, and RET* were evaluated using Sanger technology. Later, germline variants were analyzed by Next Generation Sequencing (NGS) using a custom-targeted panel of 15 genes, previously identified as driver genes: *SDHA, SDHB, SDHC, SDHD, SDHAF2, VHL, MAX, TMEM127, RET, EPAS1, FH, EGLN1, KIF1B*β*, SLC25A11, and MDH2*.

### Questionnaires

Posted questionnaires to assess speech and swallowing functions and quality of life (QoL) were administered to the entire cohort, although only a portion completed them. Speech functioning was assessed using the Voice Handicap Index (VHI) [18], and swallowing was assessed using the M.D. Anderson Dysphagia Inventory (MDAD) [19] and the Deglutition Handicap Index (DHI) [20]. The QoL was assessed by sending by email the European Organization for Research and Treatment of Cancer Quality of Life Questionnaire Head and Neck Module (EORTC QLQ-H&N35) [21].

The VHI questionnaire is a self-administered tool assessing the severity of speech disorders in three domains: functional, emotional, and physical. In every domain, the answers to different items were scored using a 0–4 Likert scale. The sum of the scores defines the speech disability as mild (<20 points), moderate (21–30 points), and severe (>30 points).

The MDADI is a self-administered questionnaire assessing the impact of dysphagia on the QoL of patients affected by head and neck tumors. This questionnaire assesses global, functional, physical, and emotional areas. The answers to every item were given a score between 1 and 5. The sum of the scores defines the deglutition function of the patients as unsatisfactory (<41 points), moderately (41–70 points), and highly satisfactory (>70 points).

The DHI questionnaire is a self-administered questionnaire consisting of 30 items on deglutition-related aspects of daily life (5-point rating scale). It is subdivided into three domains of 10 items: physical (symptoms), functional (nutritional and respiratory consequences), and emotional (psychosocial consequences). A score between 0 and 4 was given to every item of this questionnaire; the sum of the scores defines the deglutition disability as mild (<20 points), moderate (21–30 points), and severe (>30 points).

The EORTC QLQ H&N 35 is a questionnaire dedicated to patients affected by head and neck tumors, evaluating different QoL aspects of daily life related to head and neck symptomatology. It is composed of seven scales (pain, swallowing, sense, speech, social eating, social contact, and sexuality) and 11 single items that the authors considered the most important clinical aspects of QoL in head and neck cancer patients [22].

Note that only a subset of patients returned the posted questionnaires (N 32/72, 44.4%).

### Clinical assessment

All patients were offered an evaluation by an expert otolaryngologist at AOU Careggi to assess the inferior cranial nerve function (VIII, IX, X, XI, XII cranial nerves, inferior laryngeal nerve, and superior laryngeal nerve) by physical examination and fiberoptic laryngoscopy. Considering that the majority of patients were not residents of our region, we obtained an otolaryngologist (ENT) visit from a small subset of them (N 28/72, 39%).

### Statistical analysis

Statistical analysis was conducted using IBM SPSS Statistic software version 27.0 (SPSS Inc., Chicago). Descriptive statistics were performed using frequencies and percentages for categorical variables, means and standard deviation for continuous variables with parametric distributions, and median and first-third quartiles for continuous variables with a non-parametric distribution. An assessment of the normality of the distribution was made with the Shapiro-Wilk W test. Between-group differences of independent non-parametric variables were evaluated using the Mann-Whitney U test. Between-group differences of independent parametric variables were evaluated using the t-test. Between-group differences in frequency for categorical variables were evaluated using Pearson’s χ^2^ test or Fisher’s exact test. Logistic regression analysis was performed to verify significant associations. *p* values < 0.05 were considered statistically significant. A ROC curve analysis was conducted to assess the accuracy of the tumor dimension in predicting a cranial nerve lesion after surgery.

## Results

### Cohort features

We enrolled 72 patients, 47 females (65.3%) and 25 males (34.7%), with a mean age at diagnosis of 47.15 ± 16.24. The patients were divided into 2 groups: group A (patients who underwent surgery: n = 49, 68.1%) and group B (patients who underwent follow-up: N 23, 31.9%). No patients before surgery reported any cranial nerve palsy or difficulty in speech or swallowing.

Table 2 summarizes the relevant anamnestic characteristics. Comparing patients from groups A and B, patients from group A were younger (p = 0.036), and in this group, the diagnosis was more frequently made due to the onset of cervical mass (p = 0.008). Finally, patients undergoing more than one surgical intervention presented more often in familial forms (p = 0.045). Instead, patients from group B were more often asymptomatic (p = 0.042) or presented inferior cranial nerves dysfunction (p = 0.045).

**Table 2:** Anamnestic characteristics of the study population considering the two groups (group A and group B). Mean ± SD

	Group A (patients n = 49)	Group B (patients n = 23)	*p* value
Age at diagnosis (years) (mean ± SD)	44.16 ± 15.21 (n = 45)	53.57 ± 16.88 (n = 21)	**0.036**
Gender n F/M (%)	34/15(69.4/30.6)	13/10(56.5/43.5)	0.302
Symptom at diagnosis (%)			
Cervical mass	25 (51.0%)	6 (26.1%)	**0.008**
Inferior cranial nerves dysfunction	0 (0.0%)	3 (13.0%)	**0.045**
Pulsatile tinnitus	1 (2.0%)	0 (0.0%)	1.000
No available data	9 (18.4%)	0 (0.0%)	
Reason of diagnosis (n, %)			
Incidentally detected	12 (24.5%)	10 (43.5%)	0.411
Familial screening	2 (4.1%)	4 (17.4%)	0.179
Symptoms	26 (53.1%) 9	9 (39.1%)0	**0.042**
No available data	(18.4%)	(0.0%)	
Number of HNPGLs per patient (one *versus* more than one)			0.435
Number of HNPGLs per patient			
1	29 (59.2%)	15 (65.2%)	
2	12 (24.5%)	7 (30.4%)	
3	8 (16.3%)	1 (4.3%)	
Sporadic/inherited form (%)	37/49(75.5%)	12/23(52.2%)	**0.045**

bold values for *p* < 0.05

In a binary regression model with the presence of any symptoms (yes/no) as a dependent variable and adjusted for genetic results (positive/negative) and multiple HNPGLs (yes/no), older patients were more frequently affected by incidentally detected, asymptomatic HNPGLs (OR = 0.961 CI95% 0.925–1.000, p = 0.049) (Table 3).

**Table 3:** Binary adjusted regression model using the presence of any symptoms as dependent variable and age at diagnosis, multiple HNPGLs, and results of genetic analysis

	B	*P* value	OR	Lower	Upper
Age at diagnosis	−0.0.39	**0.049**	0.961	0.925	1.000
Multiple HNPGLs	−0.288	0.663	0.750	0.250	2.737
Results of genetic analysis	0.047	0.949	1.049	0.243	4.526

bold values for *p* < 0.05

A total of 76 HNPGLs were diagnosed in patients of group A: 65 carotid body tumors (85.5%), 5 vagal PGLs (6.6%), 4 jugulo-tympanic PGLs (5.3%), 1 laryngeal PGL (1.3%), and 1 not specified HNPGL (1.3%). In group B, 32 HNPGLs were diagnosed: 25 carotid body tumors (78.1%), 6 jugulo-tympanic PGLs (18.8%), and 1 vagal PGL (3.1%). No significant difference was observed between group A and group B in the localization or the maximum diameter of the HNPGLs (group A: 30.0 mm [20.0–35.0] *vs*. group B: 30.0 mm [20.0–44.0], p = 0.918) (Table 4). In group A, three patients (6.1%) underwent surgery two times and one (2.0%) underwent surgery three times.

**Table 4:** Characteristics of all HNPGLs in the study population divided in group A and group B. Median [quartiles]

	Group A (n = 76)	Group B (n = 32)	*p* value
Localisation (n, %)			
Carotid body	65 (85.5%)	25 (78.1%)	0.458
Jugulo-tympanic	4 (5.3%)	6 (18.8%)	0.381
Vagal	5 (6.6%)	1 (3.1%)	0.546
Laryngeal	1 (1.3%)	0	1.000
Unspecified	1 (1.3%)	0	
Maximum diameter (mm)	30.0 [20.0–35.0]	30.0 [20.0–44.0]	0.918

Six out of 49 patients in group A (12,2%) (two pheochromocytomas, two abdominal PGLs, one pelvic PGL, and one thorax PGL) and five out of 23 patients in group B (21.7%) (two pheochromocytomas, two thorax PGLs, and one abdominal PGL) presented associated secreting PGLs. All patients with associated secreting lesions presented pathogenetic variants of *SDHD* gene and a noradrenergic biochemical phenotype.

### Genetic data

The genetic analysis results are shown in Table 5; a significant difference between group A and group B was present considering germline variants [group A N 37 (75.5%) *vs*. group B N 12 (52.1%), p = 0.04]. All patients with an *SDHB* germline variant have been included in group A, as they were surgically treated because of the high metastatic risk. As expected, patients affected by familial forms presented more often with additional HNPGLs after surgery, compared with wild-type patients (N 16/36, 44.4% *vs. N* 0/9, OR = 1.450 CI95% 1.1136–1.851, p = 0.011) and were surgically treated more than once (p = 0.009). Furthermore, patients with pathogenic variants more often presented associated secreting lesions as compared to wild-type ones (N 11/49, 22.4% *versus* N 0/23, p = 0.01) and, therefore, underwent several surgeries for both HNPGLs and associated secreting lesions (p = 0.015).

**Table 5:** Results of genetic test in group A and group B

	Group A n = 49 n (%)	Group B n = 23 n (%)
*SDHD*	27 (55.1%)	11 (47.8%)
*SDHB*	7 (14.3%)	0 (0.0%)
*SDHC*	2 (4.1%)	0 (0.0%)
*VHL*	1 (2.0%)	0 (0.0%)
*RET*	0 (0.0%)	1 (4.3%)
*WT*	12 (24.5%)	11 (47.8%)

### Posted questionnaires

Thirty-four patients from group A [30 carotid body tumors (88.2%), one vagal PGL (2.9%), one laryngeal PGL (2.9%), and two jugulo-tympanic PGLs (5.9%)] and 18 from group B [16 carotid body tumors (88.9%), and two jugulo-tympanic PGLs (11.1%)] completed the self-assessment questionnaires related to speech and swallowing. The results of the questionnaires showed no statistically significant difference between groups in MDADI (p = 0.391) and in the EORTC QLQ H&N35 (completed by 25 patients from group A and seven from group B). Differently, statistically significant differences were found in the results of VHI and DHI (p = 0.001 and p = 0.02, respectively), with higher scores in group A (DHI: group A = 3.0 [1.0–13.5] *vs*. group B = 0.5 [0.0–4.0]; VHI: group A 8.0 [0.0–32.0] *vs*. group B 0.0 [0.0–2.0]), as shown in Table 6. The evaluation of group A was performed after a mean time from surgery of 14.38 ± 8.32 months while the evaluation of group B was performed after a mean time from diagnosis of 125.33 ± 72.27 months. Considering only patients presenting only one HNPGL who underwent surgery once [group A N 20 (40.8%): 19 carotid body tumors (95.0%) and one vagal PGL (5.0%); group B N 11 (47.8%): 9 carotid body tumors (81.8%) and two jugulo-tympanic PGLs (18.2%)], only VHI scoring maintained a significant difference between groups (group A 7.5 [0.0–25.5] *vs*. group B 0.0 [0.0–2.0], p = 0.016) while DHI scoring did not (group A 3.0 [0.0–9.0] *vs*. group B 2.0 [0.0–9.0], p = 0.525).

**Table 6:** Results of questionnaires MDADI, VHI, DHI, and EORTC QLQ – H&N35 scales comparing group A and group B

	Group A (n = 34, 69.4%)	Group B (n = 18, 78.3%)	*p-*value
MDADI	92.0 [79.2–96.0]	96.0 [77.0–96.0]	0.391
VHI	8.0 [0.0–32.0]	0.0 [0.0–2.0]	**0.001**
DHI	3.0 [1.0–13.5]	0.5 [0.0–4.0]	**0.020**

	Group A (n = 25, 51%)	Group B (n = 7, 30.4%)	*p-*value
EORTC QLQ – H&N35 scales (%)			
HN Pain	8.3 [0.0–16.7]	8.3 [0.0–16.7]	0.666
HN Swallowing	0.0 [0.0–16.7]	0.0 [0.0–8.3]	0.558
HN Senses	0.0 [0.0–0.0]	0.0 [0.0–0.0]	0.630
HN Speech	11.1 [0.0–16.6]	0.0 [0.0–11.1]	0.120
HN Social eating	0.0 [0.0–0.0]	0.0 [0.0–0.0]	0.640
HN Social contact	0.0 [0.0–0.0]	0.0 [0.0–0.0]	0.176
HN Sexuality	0.0 [0.0–25.0]	0.0 [0.0–0.0]	0.096
HN Teeth	0.0 [0.0–16.7]	0.0 [0.0–0.0]	0.637
HN Opening mouth	0.0 [0.0–0.0]	0.0 [0.0–0.0]	0.233
HN Dry mouth	0.0 [0.0–0.0]	0.0 [0.0–33.3]	0.083
HN Sticky saliva	0.0 [0.0–0.0]	0.0 [0.0–33.3]	0.067
HN Coughed	0.0 [0.0–33.3]	33.3 [0.0–33.3]	0.932
HN Felt ill	0.0 [0.0–16.7]	0.0 [0.0–0.0]	0.473
HN Pain killers	0.0 [0.0–100.0]	0.0 [0.0–0.0]	0.280
HN Nutritional supp.	0.0 [0.0–0.0]	0.0 [0.0–0.0]	0.173
HN Feeding tube	0.0 [0.0–0.0]	0.0 [0.0–0.0]	0.573
HN Weight loss	0.0 [0.0–0.0]	0.0 [0.0–0.0]	0.699
HN Weight gain	0.0 [0.0–0.0]	0.0 [0.0–0.0]	0.233

In MDADI the higher the result less is the disability. In VHI, DHI and EORTC QLQ-H&N35 higher the result higher the disability. Median [quartiles]

bold values for *p* < 0.05

At ROC curve analysis, a VHI score ≥4 significantly identified patients undergoing surgery (group A) from those who did not (group B) with a sensibility and specificity of 67.6 and 88.9%, respectively (AUC 0.77 ± 0.064 CI95% 0.644–0.895, p < 0.0001). In a confounder-adjusted (age at diagnosis and genetic result) binary regression model, having a VHI ≥ 4 (mild vocal impairment during the follow-up) is associated with a higher rate of any clinical symptoms at diagnosis (OR = 4.720 CI95% 1.055–21.108, p = 0.042) and a higher number of surgical interventions throughout life (OR = 3.580 CI95% 1.093–11.725, p = 0.035) (Table 7).

**Table 7:** Binary regression model using a VHI ≥ 4 as dependent variable and results of genetic analysis, presence of any symptoms at diagnosis, and number of head and neck surgical interventions

	B	*P* value	OR	Lower	Upper
Age at diagnosis	−0.024	0.419	0.977	0.922	1.034
Results of genetic analysis (negative/positive)	−1.259	0.217	0.284	0.039	2.092
Symptoms (no/yes)	1.552	**0.042**	4.720	1.055	21.108
Number of head and neck surgical interventions	1.275	**0.035**	3.580	1.093	11.725

bold values for *p* < 0.05

### ENT evaluation

Eighteen patients in group A (36.7%) and ten patients in group B (43.5%) accepted the ENT evaluation. We found a higher prevalence of cranial nerve lesions in patients of group A *versus* patients of group B (N 9/18 *vs*. N 0/10) (p = 0.007). The number and localization of the nerve lesions in group A are described in Table 8.

**Table 8:** Number and localization of the nerve lesions in group A

	Group A (n 18)
Number of patients with nerve lesion (n, %)	9 (50.0%)
Number of nerve lesions per patient (%)	
No lesions	9 (50.0%)
1	5 (27.8%)
2	3 (16.7%)
3	0 (0.0%)
4	1 (5.6%)
Number of nerve lesions per site (%)	
VIII cranial nerve	0 (0.0%)
IX cranial nerve	1 (5.6%)
X cranial nerve	1 (5.6%)
XI cranial nerve	0 (0.0%)
XII cranial nerve	2 (11.1%)
Superior laryngeal nerve	1 (5.6%)
Inferior laryngeal nerve	9 (50.0%)

Considering the nine patients with nerve lesions, two undergoing surgery twice (22.2%) and one three times (11.1%), 5/9 (55.5%) presented more than one HNPGL, and 7/9 (77.8%) presented a hereditary form.

### Sub-analysis of carotid body tumors

**Fig. 1:**
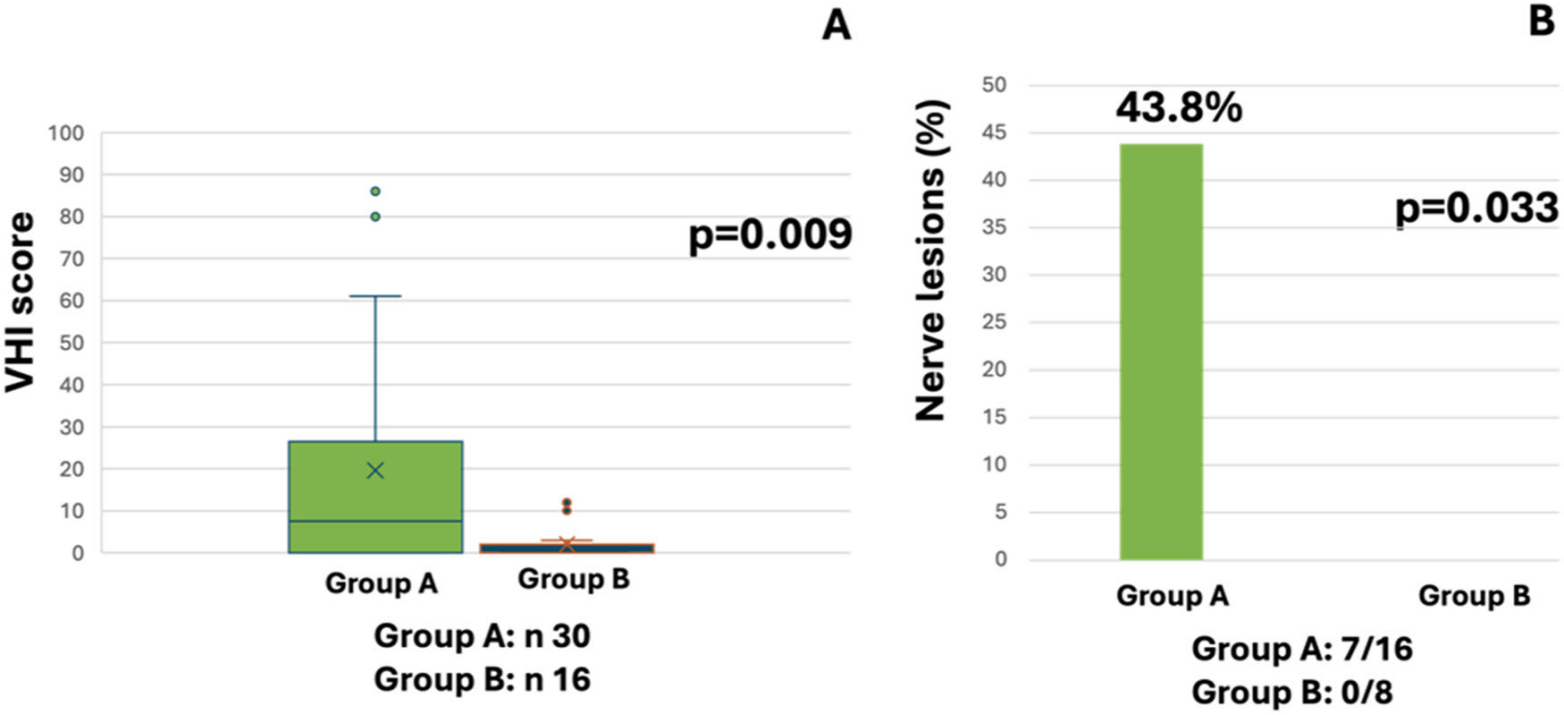
Sub-analysis in carotid body tumors. **A** Results of VHI self-assessment questionnaire in group A and B (p = 0.009); **B** results of otolaryngologist evaluation in both groups (p = 0.033)

A sub-analysis regarding only patients affected by carotid body tumors was then conducted (N 30 in group A, and N 16 in group B). VHI scoring was higher in group A than in group B, p = 0.009 (Fig. 1A). No significant differences were also observed in MDADI scoring (p = 648) as well as in all the items of the EORTC QLQ – H&N35 scale. At the ENT evaluation, seven out of 16 people in group A (43.8%) and no one in group B (N 0/8) presented cranial nerve lesions (p = 0.033) (Fig. 1B).

Finally, we performed ROC curve analysis to identify the maximal diameter of the carotid body tumor associated with any cranial nerve injuries, as assessed by ENT evaluation. ROC curve analysis on 13 carotid body tumors undergoing surgery only once (median diameter 31.0 mm [27.0–37.5]), although without reaching statistical significance, showed that a diameter greater than 34 mm can predict cranial nerve injury (AUC 0.750 ± 0.142 p = 0.078).

## Discussion

The present study aims to shed light in the context of the difficult therapeutic management of HNPGLs. Present results suggest that for smaller tumors, a surgical approach is less often enriched in post-surgical neurological complications, while, for tumors greater than 3.4 cm, they are often present and require adequate counseling because other options such as active surveillance (“wait-and-see”) could also be considered. In the present cohort, active surveillance was more often chosen in asymptomatic, older patients in whom the tumor was incidentally diagnosed. Having a genetic footprint also suggests a wait-and-see approach due to the possibility of recurrent tumors, even outside the head and neck location, including secreting forms. Scoring higher than four in voice disability (VHI) increases the risk of undergoing multiple surgical interventions by almost fourfold.

It is worth mentioning that HNPGLs are rare, slow-growing tumors, frequently close to important vasculo-nervous structures in the head and neck, potentially causing morbidity due to compression or injury of these structures. Furthermore, more than 30% of HNPGLs are familial form, with the underlying risk of developing multiple lesions lifelong. For these reasons, follow-up and therapy of these patients represent a challenge for clinicians, and a personalized evaluation is mandatory. Therefore, before any therapeutic choice is made, it is essential to evaluate multiple aspects regarding either the characteristics of the lesion (growth speed, size, location, and results of nuclear medicine exams) or of the patient (germline variants, morbidity, and age).

Our study demonstrates that patients with germline variants present multiple HNPGLs and PPGLs and undergo a greater number of surgeries. While PPGLs in most cases are secreting lesions that must be removed, HNPGLs are often not. It should therefore be carefully investigated whether, in a patient at risk of developing other lesions, the surgical option still represents the best therapeutic option. Genetic analysis is recommended in all patients affected by PPGLs [23]. Our data confirm that in patients with HNPGLs, the genetic results can significantly change therapeutic management.

While surgery has historically represented the treatment of choice, to date, therapeutic possibilities include active surveillance (“wait-and-see” approach) or radiotherapy such as EBRT and SRS (Cyberknife and Gammaknife).

Considering that maximal HNPGL diameters were similar at study entry in those undergoing (Group A) or not (Group B) a surgical approach, it is interesting that we found a higher scoring in speech and deglutition disabilities in those in Group A. This is tantamount to saying that the growth of the tumor itself (as observed in Group B) is less often responsible than the surgical intervention (Group A) for these disabilities. Hence, the possibility of active surveillance should be discussed with these patients, in particular those with the largest tumors.

Literature data report increasing risks of surgery-related complications according to the size of the lesion. For carotid body PGLs, the Shamblin class also correlates with the risk of postoperative complications [24, 25]. The complications can affect the lower cranial nerves (VII, IX, X, XI, and XII), while cerebrovascular complications (transient ischemic attacks, stroke, and hemorrhage) are less often observed (0–4%) [25–28]. Numerous retrospective studies reported that the incidence of post-surgery cranial nerve deficits is higher closer to surgery (19–42.1%), being permanent in a lower percentage of patients (0–24%) [25, 28]. Few studies describe the long-term outcomes of the surgery of head and neck PGLs. A meta-analysis shows that in carotid body tumors, the risk of cranial nerve injuries was higher as a function of higher Shamblin classes (up to 30% in Shamblin III), but there was a high level of variability in the included studies [29]. A systematic review of jugular and vagal PGLs conducted by Suarez et al. showed an 181.5% increase in cranial nerve palsies in patients surgically treated [30]. Accordingly, our data show a higher prevalence of cranial nerve damage in patients surgically treated, as derived either from self-reported questionnaires (VHI, DHI) or from ENT evaluation. Patients treated with surgery presented a significantly higher DHI and VHI total score, suggesting the presence of swallowing and speech disabilities, although to a mild extent. In group A the greater percentage of vagal PGLs compared with group B (6.6 versus 3.1%) could partly justify the higher presence of nerve injury. In the available ENT evaluations, we demonstrated the presence of nerve lesions in almost 50% of cases, a higher frequency as compared to previous studies [31] which showed a percentage of 13.9%, which rises to 24.5% if transient lesions are also included. This difference may be linked to the larger median tumor diameter of the present study than in a previous one, i.e., 3 *vs*. 2.5 cm, or to the lack of long-term follow-up in the present study [31]. In patients affected by carotid body tumors treated with surgery, ROC curve analysis shows a tendency towards an increased risk of nervous damage when the HNPGLs exceed the maximum dimension of 3.4 cm. However, QoL seems to be the same between group A and group B.

Bearing in mind that surgical excision is the only curative treatment option, our results highlighted that surgery still represents the first option for patients with small HNPGLs due to the lower risk of associated complications. Furthermore, surgery also represents the first choice in symptomatic tumors and in those with rapid growth, due to the risk of compression of the adjacent vascular-nervous structures. Moreover, herein we report that after surgery, in patients with larger lesions, only moderate speech and swallowing deficits were found, without significant difference in QoL, as previously reported [32].

Radiotherapy can be proposed as a primary treatment for large tumors, as a palliative treatment in the case of post-surgical recurrence [33], and in patients in whom surgery is contraindicated due to poor general condition. A recent meta-analysis [34] showed that SRS has a local tumor control rate of 94% against 81.3% of surgery and a 78% higher probability of achieving local tumor control than surgery alone, with a lower risk of morbidity.

Nevertheless, considering that HNPGLs are tumors that are mostly indolent and slow-growing, active surveillance (“wait-and-see”) should also be considered as a first option. This approach is preferable in asymptomatic patients with multiple tumors, familial forms, and a slow growth rate. In patients included in group B, the long time elapsed between diagnosis and evaluation supports the hypothesis that the growth over time of these lesions rarely leads to develop neural deficits. Otherwise, the missing of an earlier evaluation at the time of diagnosis does not permit to exclude the presence of a pre-existing nerve impairment although no one presented related signs and symptoms. Accordingly recent consensus guidelines on the treatment of *SDHD* [35] and *SDHB* [36] PPGLs suggest initial active surveillance in non-metastatic HNPGLs.

Our results also demonstrate that older patients are more frequently affected by asymptomatic HNPGLs. Therefore, considering the expected slow growth of these tumors, they should be candidates for a wait-and-see approach.

Regarding quality of life (QoL), in 2013, van Hulsteijn LT et al., [32] evaluated the QoL in 174 patients affected by HNPGLs and/or *SDHx* mutations versus controls. They reported a decrease in QoL score considering physical and psychological aspects due to the presence of signs and symptoms caused by nerve lesions and not to the HNPGL per se. The authors also reported the stability of QoL over time, the absence of differences between wild-type and *SDHx* patients, and between patients affected by different germline variants, such as the highest-risk mutation *SDHB*. Furthermore, surgery did not affect QoL. Our results keep showing no major difference for QoL in those undergoing or not undergoing surgery. The mild nature of surgery for VHI- and DHI-associated disabilities could underscore this result.

Our study presents some limitations: I) the small number of included patients; II) the heterogeneity of the population; III) the higher prevalence of carotid body tumors; IV) the absence of a dedicated ENT visit in the entire study population; V) the impossibility of carrying out sub-analyses considering the different localizations of the HNPGLs; VI) genetic analysis of a limited number of genes in patients enrolled before 2017, and VII) the absence of a long-term follow-up. In fact, a longer follow-up starting from the diagnosis could permit to recognize the improvement of symptoms due to speech therapy and rarely corrective surgery and the possibility of worsening of clinical status. Finally, the lack of completed questionnaires and ENT evaluation in the entire cohort is another important limitation that can determine a selection bias. For these reasons, more studies are needed to fully understand the best treatment for HNPGLs and the long-term efficacy of surgery as compared to non-surgical treatments. In particular, a study dedicated to lesions with different localizations (vagal, jugulo-tympanic, and laryngeal PGLs) is necessary, considering the different risks of metastases which reaches up to 19% in patients with vagal PGLs [37].

In conclusion, as emerged from the First International Congress on Head and Neck Paragangliomas in 2023 [6], a closer collaboration between healthcare specialists who take care of patients affected by HNPGLs is necessary. Indeed, surgery should be considered a first-line treatment in younger patients with small HNPGLs, and therefore, the clinicians must request a surgical opinion during the first assessment. However, a clinical evaluation is mandatory before surgery to perform the genetic analysis and screening for associated secreting lesions to avoid non-resolving surgery and possible neurovascular complications.

## Data Availability

No datasets were generated or analysed during the current study.
